# Novel Aurora A Kinase Inhibitor Fangchinoline Enhances Cisplatin–DNA Adducts and Cisplatin Therapeutic Efficacy in OVCAR-3 Ovarian Cancer Cells-Derived Xenograft Model

**DOI:** 10.3390/ijms23031868

**Published:** 2022-02-07

**Authors:** Daniel Winardi, Pei-Yi Chu, Guan-Yu Chen, Ke Wang, Wei-Yu Hsu, Ching-Liang Hsieh, Yung-Hsiang Chen, Yang-Chang Wu, Juan-Cheng Yang

**Affiliations:** 1 Graduate Institute of Integrated Medicine, College of Chinese Medicine, China Medical University, Taichung 404, Taiwan; hatasjkimo@yahoo.com.tw (D.W.); clhsieh@mail.cmuh.org.tw (C.-L.H.); yhchen@mail.cmu.edu.tw (Y.-H.C.); 2 Chinese Medicine Research and Development Center, China Medical University Hospital, Taichung 404, Taiwan; ositachucmu@gmail.com (P.-Y.C.); markchen19822001@gmail.com (G.-Y.C.); waker603@163.com (K.W.); oscar_c20@hotmail.com (W.-Y.H.); 3 Sex Hormone Research Center, Department of Obstetrics and Gynecology, China Medical University Hospital, Taichung 404, Taiwan; 4 Department of Chinese Medicine, China Medical University Hospital, Taichung 404, Taiwan; 5 Department of Medical Laboratory Science and Biotechnology, College of Medical and Health Science, Asia University, Taichung 413, Taiwan; 6 Department of Psychology, College of Medical and Health Science, Asia University, Taichung 413, Taiwan; 7 School of Chinese Medicine, College of Chinese Medicine, China Medical University, Taichung 406, Taiwan

**Keywords:** Aurora A kinase, inhibitor, fangchinoline, cisplatin, ovarian cancer, mice

## Abstract

Aurora A kinase (Aurora A) is a serine/threonine kinase regulating control of multiple events during cell-cycle progression. Playing roles in promoting proliferation and inhibiting cell death in cancer cells leads Aurora A to become a target for cancer therapy. It is overexpressed and associated with a poor prognosis in ovarian cancer. Improving cisplatin therapy outcomes remains an important issue for advanced-stage ovarian cancer treatment, and Aurora A inhibitors may improve it. In the present study, we identified natural compounds with higher docking scores than the known Aurora A ligand through structure-based virtual screening, including the natural compound fangchinoline, which has been associated with anticancer activities but not yet investigated in ovarian cancer. The binding and inhibition of Aurora A by fangchinoline were verified using cellular thermal shift and enzyme activity assays. Fangchinoline reduced viability and proliferation in ovarian cancer cell lines. Combination fangchinoline and cisplatin treatment enhanced cisplatin–DNA adduct levels, and the combination index revealed synergistic effects on cell viability. An in vivo study showed that fangchinoline significantly enhanced cisplatin therapeutic effects in OVCAR-3 ovarian cancer-bearing mice. Fangchinoline may inhibit tumor growth and enhance cisplatin therapy in ovarian cancer. This study reveals a novel Aurora A inhibitor, fangchinoline, as a potentially viable adjuvant for ovarian cancer therapy.

## 1. Introduction

Ovarian cancer, the fifth cause of cancer-related deaths among women in the United States [1], leads to 152,000 deaths annually in the world [2]. Because the symptoms are not obvious during early stages, approximately 70% of patients are diagnosed with ovarian cancer at advanced stages, with poor prognosis [3]. The five-year relative survival rate is approximately 45%. The standard treatment for ovarian cancer consists of cytoreductive surgery, followed by cisplatin-based chemotherapy [4]. Although cisplatin is effective, it is associated with undesirable side effects, including severe kidney problems, allergic reactions, decreased immunity to infections, gastrointestinal disorders, and hemorrhage, and cancer resistance remains a clinical complication in advanced-stage patients [4,5]. Developing new agents to improve ovarian cancer therapy remains an urgent issue. Combination therapy is one strategy that has been pursued to overcome these problems, and cisplatin-based combination chemotherapy has improved some therapeutic efficiencies in the clinic [6].

Aurora A kinase (Aurora A) is an enzyme in the Aurora kinase serine/threonine family of proteins, which play critical roles in the cell cycle [7]. In normal cells, Aurora A mainly accumulates and becomes activated in the G2 phase and becomes inactivated and degraded at the metaphase–anaphase transition [8]. However, in various cancer tissues, the expression of Aurora A is overexpressed, regardless of their cell-cycle phases. It is typically upregulated in primary neoplastic cells and several types of solid epithelial tumors, including ovarian, breast, pancreatic, colon, and bladder cancers [9,10], and Aurora A is thought to be involved in cancer pathogenesis [11,12]. By directly or indirectly affecting the phosphorylation or the expression of various proteins in signaling pathways, such as IKK kinases, IκB-α, p53, mTOR, and retinoblastoma protein, Aurora A plays multiple regulatory roles in cancer development, such as promoting cell-cycle progression, activating cell survival pathways, inhibiting apoptosis signaling, and inducing genomic instability [8]. Therefore, Aurora A has been regarded as an important target for cancer therapy. In ovarian cancer, Aurora A overexpression has been linked to a poor survival rate [13]. In addition, Aurora A is involved in drug resistance mechanisms in cancer. The inhibition of Aurora A enhances the cytotoxic effects of cisplatin in ovarian cancer cells [14], suggesting that Aurora A inhibition could improve ovarian cancer therapy. The binding protein TPX2 is a well-characterized Aurora A activator [15]. The autophosphorylation of the Thr288 residue increases the catalytic activity of Aurora A [16], and TPX2 binding prevents the dephosphorylation of p-Thr288 in the kinase activation loop, stabilizing the active conformation. Residues 1–43 of TPX2 have been reported to be sufficient for Aurora A binding, kinase activation, and protection from dephosphorylation [17]. The Y pocket and W pocket are hot spots in the Aurora A/TPX2 interaction [18,19]. The development of molecules that target the Y–W site has been adopted as a potential strategy for developing novel Aurora A inhibitors.

Natural products have been used to treat various diseases since ancient times. The compounds found in natural products are perfect resources for drug discovery, providing novel small-molecule lead compounds [20]. For example, paclitaxel, one of the most popular anticancer drugs, was initially extracted from the bark of the Pacific yew tree. To date, the proportion of natural resources that have been evaluated for biological activity is very small, and abundant potential drug compounds remain to be discovered [21]. To accelerate the drug discovery process, various computational methods have been developed to identify and analyze the ability of small-molecule ligands to interact with target proteins. Virtual screening, molecular docking, molecular dynamics simulation, and the calculation of free binding energies are popular tools [22]. GOLD software is a useful software for molecular docking. By evaluating the intermolecular hydrogen bond formation, van der Waals interactions between the protein and ligand, the prediction of the binding affinity between compounds and the target protein are presented as a goldscore [23].

In the present study, we performed structure-based virtual screening (VS) using GOLD software to identify potential Aurora A inhibitors, resulting in the discovery of 12 compounds, including fangchinoline. Fangchinoline is a bisbenzylisoquinoline alkaloid isolated from the dried root of *Stephania tetranda S. Moore*. We verified and characterized the fangchinoline-mediated inhibition of Aurora A, including anticancer effects and combination effects with cisplatin therapy, in ovarian cancer.

## 2. Results

### 2.1. Structure-Based VS and Docking

To identify natural products in the Taiwan Database of Extracts and Compounds (TDEC) database (2303 compounds; https://tdec.kmu.edu.tw, accessed on 2 December 2021) capable of binding to the Y–W site of Aurora A, structure-based VS was performed using GOLD software and the compound A4W (ligand A4W), a ligand bound in the Y-W site of AURKA (PDB ID: 5ORL), was used as a standard ligand [18]. We found that the binding score of 260 compounds docking into the Y-W site was better than the ligand A4W (goldscore > 49.03). We selected the 12 compounds with high binding affinities (Supplementary Table S1) to further examine their in vitro bioactivities. The half-maximal inhibitory concentration (IC_50_) against ovarian cancer cell viability was analyzed for these 12 compounds to evaluate their potential for ovarian cancer therapy (Supplementary Table S2). Compared with the other compounds, fangchinoline had the high goldscore (57.19) and the low IC_50_ (9.66 µM in OVCAR-3, 8.71 µM in MDAH 2774, 25.10 µM in ES-2, and 11.74 µM in SK-OV-3), indicating a greater potential for future applications.

**Figure 1:**
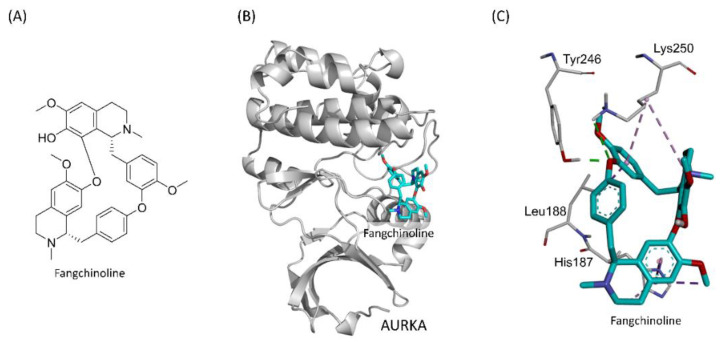
The simulation of fangchinoline docking into the Y-W site of Aurora A. The structure of fangchinoline (**A**). Fangchinoline (cyan stick) docked into the Y-W site of Aurora A (gray cartoon) (**B**). The hydrogen-bonding interactions (green dashed line) and hydrophobic interactions (purple dashed line) in the Y-W site of Aurora A were presented (**C**).

To understand the interactions between fangchinoline and Aurora A, a simulation analysis was performed using DS 2018 Visualizer software. Figure 1B shows the simulated docking of fangchinoline with the Y-W site of Aurora A, which forms two electrostatic hydrogen-bonding interactions (green dashed line) with Tyr246 (1.98 Å) and Lys250 (3.09 Å). In addition, it formed a hydrophobic alkyl–alkyl interaction (light purple dashed line) with Lys250 (5.33 Å), two pi–alkyl interactions (purple dashed line) with His187 (4.44 Å) and Lys250 (4.70 Å), and two hydrophobic pi–sigma interactions (dark purple dashed line) with His187 (3.79 Å) and Leu188 (3.01 Å). A hydrophobic pi–pi interaction (lavender dashed line) was also formed with His187 (3.46 Å) in the Y–W site of Aurora A (Figure 1C).

### 2.2. Inhibitory Effects of Fangchinoline against Aurora A

**Figure 2:**
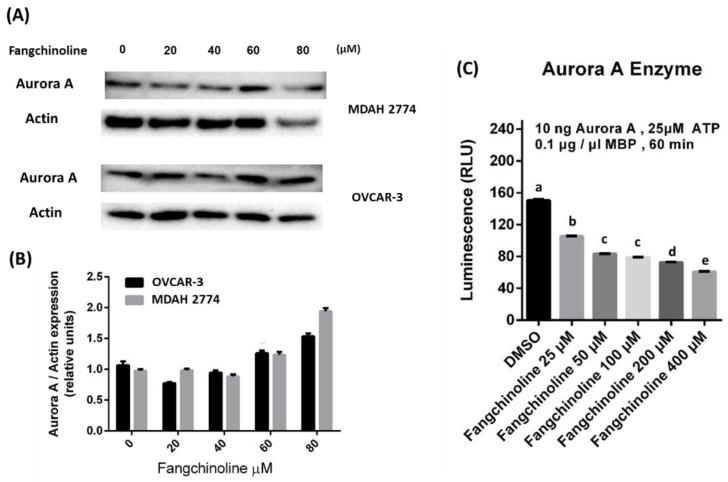
The inhibition of Aurora A enzymatic activity by fangchinoline. The binding between fangchinoline and Aurora A protein was evaluated using the cellular thermal shift assay. After the application of a heat challenge to fangchinoline-treated MDAH 2774 and OVCAR-3 cells, the level of Aurora A protein was detected by Western blot analysis (**A**), and the quantification of the Aurora A expression is presented (**B**). To investigate the inhibitory effects of fangchinoline against Aurora A enzymatic activity, fangchinoline (0, 25, 50, 100, 200, or 400 μM) was incubated with Aurora A in vitro, and the enzyme activity was determined using an assay kit. Different letters indicate statistically significant differences between groups (*p* < 0.05). Data are presented as the mean ± SD (*n* = 3) (**C**).

To confirm the binding of fangchinoline with Aurora A protein, a cellular thermal shift assay (CETSA) was performed, which allows for the evaluation of ligand–protein binding through the assessment of target protein level after heat challenge [24,25]. The binding of ligand modulates conformational and thermal stability of proteins, which causes the resistance of the protein against heat-induced denaturation. Therefore, after the compound treatment, the binding between the compound and the target protein can be evaluated by the increase in the target protein level after the heat challenge [25]. In the present study, cells were treated with fangchinoline for 1 h, followed by a heat challenge, and the Aurora A protein was detected by Western blot analysis. Figure 2A,B shows that fangchinoline treatment increased Aurora A protein levels in a dose-dependent manner after heat challenge in both MDAH 2774 and OVCAR-3 cells, suggesting that fangchinoline binding with Aurora A protein improved protein thermos-stability. To investigate the inhibitory effects of fangchinoline against Aurora A activity, Aurora A enzymatic activity was evaluated in vitro. Figure 2C shows that fangchinoline treatment significantly decreased Aurora A enzymatic activity in a dose-dependent manner. These results indicated that fangchinoline acts as a novel Aurora A inhibitor.

### 2.3. Effects of Fangchinoline on Ovarian Cancer Cell Line

**Figure 3:**
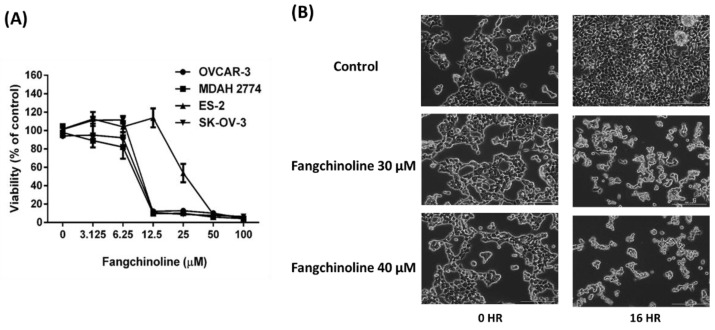
Anticancer effects of fangchinoline on ovarian cancer cells. The effects of the fangchinoline on viability were assessed in ovarian cancer cell lines OVCAR-3, MDAH 2774, ES-2, and SK-OV-3. Cells were treated with fangchinoline (0, 3.125, 6.25, 12.5, 25, 50 or 100 μM) for 48 h. Data are presented as percent of the control (fangchinoline 0 μM treatment) viability; mean ± SD (*n* = 4) (**A**). The growth of OVCAR-3 cells was observed with phase-contrast microscopy. OVCAR-3 cells were treated with fangchinoline (0, 30, or 40 μM) for 16 h. All images were obtained at a magnification of ×40 (**B**).

Although fangchinoline has been reported to inhibit various cancer types, the anticancer effects of fangchinoline in ovarian cancer have never been investigated. The effects of fangchinoline on ovarian cancer cell viability and morphology were investigated in the ovarian cancer cell lines OVCAR-3, MDAH 2774, ES-2, and SK-OV-3. Cells were treated with fangchinoline (0, 3.125, 6.25, 12.5, 25, 50, or 100 μM), and cell viability was assessed 48 h after treatment. The data showed that fangchinoline decreased ovarian cancer cell viability in a dose-response manner. When fangchinoline concentrations were greater than 12.5 μM, the viability of OVCAR-3, MDAH 2774, and SK-OV-3 cell lines was lower than 20% (Figure 3A). Morphological changes could be observed at 16 h after treatment with 30 and 40 μM fangchinoline. Cell proliferation was assessed by comparing the cell confluence at 16 h with the confluence observed at 0 h. The data showed that 30 and 40 μM fangchinoline treatments did not increase cell confluence at 16 h. In addition, morphological hallmarks of apoptosis, including the loss of cell volume and cell shrinkage, were observed after 16 h of fangchinoline treatment (Figure 3B). These results suggest that fangchinoline possesses anticancer properties against ovarian cancer.

### 2.4. Effects of Fangchinoline on Cisplatin Treatment in Ovarian Cancer Cells

**Figure 4:**
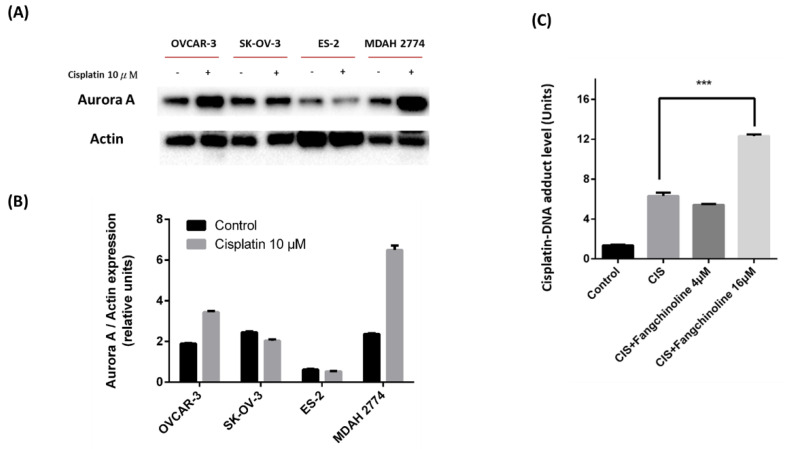
Fangchinoline enhances cisplatin efficacy in ovarian cancer cells. Aurora A expression was observed in cisplatin-treated ovarian cancer cells. The ovarian cancer cell lines OVCAR-3, SK-OV-3, ES-2, and MDAH 2774 were treated with 10 µM cisplatin for 48 h, and Aurora A expression was detected by Western blotting (**A**). The quantification of the Aurora A expression (**B**). To investigate the effects of fangchinline treatment on cisplatin–DNA adduct formation in ovarian cancer cells, OVCAR-3 cells were treated with fangchinoline and cisplatin (CIS; 10 µM) for 48 h, and cisplatin–DNA adduct levels were detected using an anti-cisplatin-modified DNA antibody, followed by flow cytometry. Data are presented as the mean ± SD (*n* = 3). *** *p* < 0.001 compared to cisplatin group (**C**).

Aurora A inhibitor has been reported to induce esophageal adenocarcinoma cell death and enhance cisplatin therapy in vivo [26]. Therefore, we further investigated the effect of fangchinoline on cisplatin treatment in ovarian cancer. It is reported that cisplatin upregulates the expression of Aurora protein in Hela cells [27]. However, the enhancing effect of cisplatin on Aurora A expression has never been investigated in ovarian cancer. To find the critical cell line for the cisplatin therapy study, we tested the effects of cisplatin on Aurora A expression in several ovarian cancer cell lines. The results showed that Aurora A expression was upregulated by cisplatin treatment in OVCAR-3 and MDAH 2774 cell lines (Figure 4A,B). Choosing OVCAR-3 could be more meaningful because OVCAR-3 is a cisplatin-refractory cell line that was established from a tumor sample obtained from a patient with progressive ovarian cancer following treatment with combination chemotherapy consisting of cyclophosphamide, adriamycin, and cisplatin [28]. Thus, we focused on OVCAR-3. Fangchinoline was added to cisplatin-treated OVCAR-3 cells. Figure 4B shows that 16 µM fangchinoline treatment significantly increased the formation of cisplatin–DNA adducts in cells. The combined effect of cisplatin plus fangchinoline treatment was analyzed by calculating a combination index (CI) value. Cisplatin/fangchinoline treatments showed synergistic effects on reduced cell viability, with CI values of 0.77 at a molar ratio of 16/1, 0.513 at a molar ratio of 1/1, and 0.78 at a molar ratio of 0.5/1 (Table 1). These data indicate that fangchinoline enhances the anticancer effects of cisplatin.

### 2.5. Effects of Fangchinoline on Cisplatin Therapy in Mice with Ovarian Cancer

**Figure 5:**
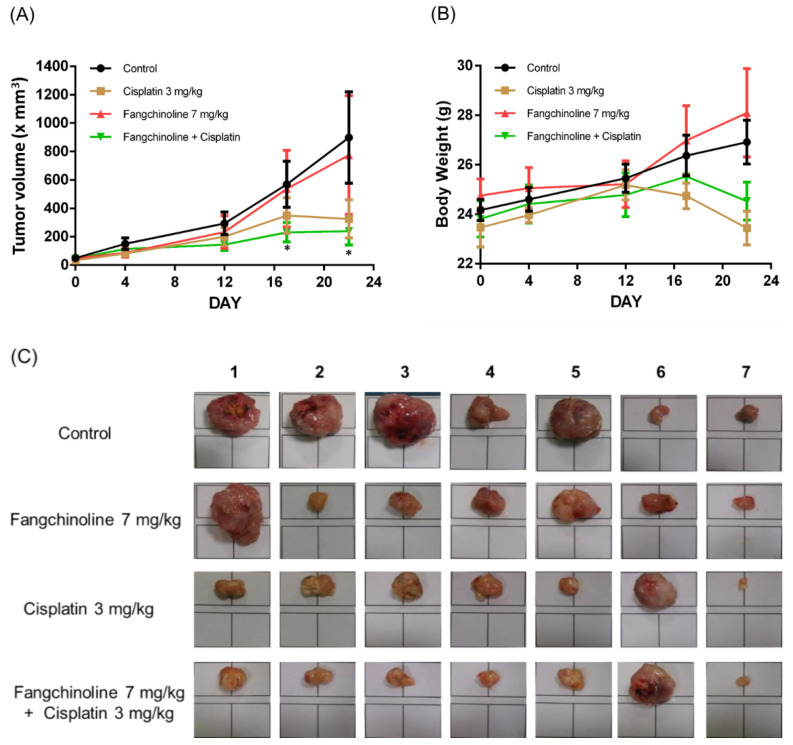
Fangchinoline enhanced the cisplatin therapeutic efficacy in ovarian tumor-bearing mice. Female NOD SCID mice were implanted subcutaneously with OVCAR-3 cells. Tumor-bearing mice were administered weekly with saline, fangchinoline, or cisplatin i.v. injection. Tumor volume (**A**) and body weight (**B**) were measured. All mice were sacrificed on Day 22, and the tumor images were taken under the same scale (**C**). Data are expressed as the mean ± SEM (*n* = 7).

**Figure 6:**
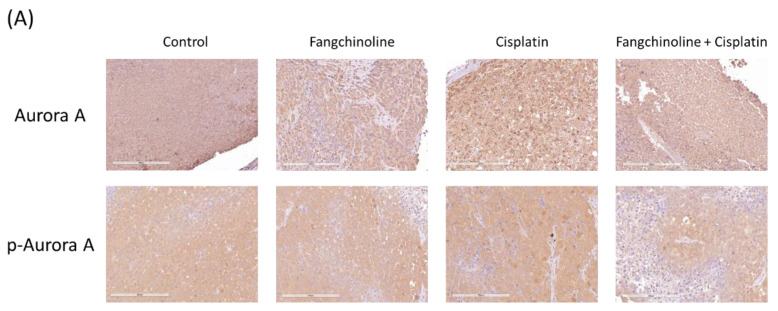

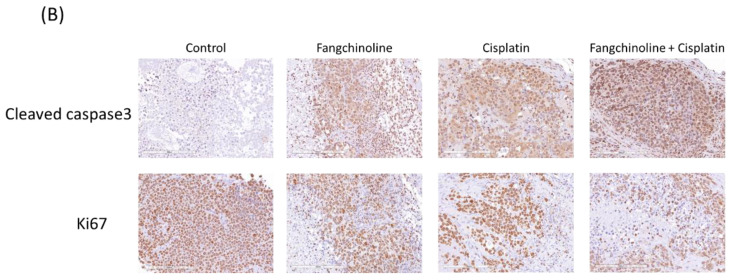
Protein detection in tumor tissue. Tumors were collected on Day22. Aurora A activation was observed by detecting Aurora A and phospho (p)-Aurora A protein expressions in tumor tissue (**A**). Apoptosis and proliferation were evaluated by detecting cleaved caspase 3 and Ki67 protein expressions, respectively (**B**). Immunohistochemical staining was performed. Proteins were observed with brown staining. Original magnification, ×100. Scale bar: 200 μm.

To study the effects of fangchinoline on cisplatin therapy in vivo, combined cisplatin and fangchinoline treatment was given to mice bearing ovarian cancer tumors. The cancer model was established through the subcutaneous injection of OVCAR-3 cells into non-obese diabetic severe combined immunodeficiency (NOD SCID) mice. Cisplatin, at 3 mg/kg, and fangchinoline, at 7 mg/kg, were administered weekly to ovarian cancer mice. Tumor volumes and body weights were measured. The data show that treatment with 3 mg/kg cisplatin significantly inhibited ovarian tumor growth in mice. Combination treatment with 7 mg/kg fangchinoline and 3 mg/kg cisplatin enhanced the anticancer effects in mice compared with cisplatin treatment alone (Figure 5A) without affecting body weight (Figure 5B). Figure 5C shows images of the tumors on Day 22. Data show that fangchinoline enhanced the cisplatin treatment on controlling tumor growing speed. The in vivo validation of Aurora A inhibition was assessed by detecting Aurora A and p-Aurora A expression. Aurora A and p-Aurora A expressions in tumor tissue were increased by cisplatin and decreased in cisplatin plus fangchinoline group (Figure 6A). In addition, fangchinoline enhanced the effect of cisplatin on apoptosis and proliferation. The expression of the apoptosis marker, cleaved caspase 3, was increased, and the proliferation marker, Ki67, was decreased in the fangchinoline plus cisplatin group compared to the cisplatin group (Figure 6B). It seems that fangchinoline is beneficial in enhancing cisplatin efficacy in ovarian cancer therapy.

## 3. Discussion

Structure-based VS using the molecular docking methodology has been broadly applied for the purposes of drug discovery for over a decade [29,30]. To identify potential small molecules able to bind the active site of a target protein, many useful VS docking tools have been developed, such as DOCK, AutoDock Vina, and GOLD [30]. The literature suggests that GOLD performs reasonably well in VS studies for predicting the binding poses between small-molecule compounds and the active sites of target proteins, with a suitable hit rate [30]. GOLD uses a genetic algorithm (GA) to search for reasonable compound conformations and predict reliable binding models between identified compounds and the target protein [31], identifying correct ligand binding poses with 90.0% accuracy [32]. The goldscore is one scoring function that can be obtained using GOLD software, which has displayed suitable performance for predicting the binding affinity between compounds and protein [23]. The goldscore scoring function considers several terms, including H-bonding energy, van der Waals energy, metal interaction, and ligand torsion strain [23]. Furthermore, the reliability of GOLD was verified by the evaluation of the PDBbind database, which includes 1300 protein complexes, resulting in fewer than 30 failed complexes [33]. Over the years, GOLD has been verified in the search for active inhibitors against several targets, including human apurinic/apyrimidinic endonuclease 1 [34], glycogen synthase kinase (GSK)-3β kinase [35], and ricin [36]. In the present study, we applied GOLD software using GA to investigate the binding between natural compounds in the TDEC database and the active site (Y–W site) of the Aurora A protein. The Y–W site is an allosteric site for binding with the TPX2 modulator, which facilitates the active conformation of Aurora A [19]. The development of Aurora A inhibitors has the potential to improve cancer therapy by regulating cancer cell growth, inducing cancer cell death, attenuating drug resistance, and enhancing chemotherapy [37,38,39].

Fangchinoline is a natural compound extracted from the root of *Menispermaceae* family members, such as *Stephania tetrandra S. Moore* and *Cyclea peltata Diels.* The herb, known as Fang Ji, is a traditional Chinese medicine [40]. Fangchinoline, which is a bisbenzylisoquinoline alkaloid, is the main bioactive compound in Fang Ji and possesses various functions, including anticancer, anti-inflammatory, antioxidant, anti-osteoporosis, and neural protective effects [41]. Fangchinoline inhibits cancer proliferative activity, cell migration activity, and tumor cell growth and induces apoptosis [40]. However, to date (17 July 2021), the anticancer effects of fangchinoline remain poorly understood, and only 43 journal articles discussing fangchinoline were identified in the PubMed database (https://pubmed.ncbi.nlm.nih.gov/, accessed on 2 December 2021). Although many cancer types are inhibited by fangchinoline, including breast, bone, lung, melanoma, leukemia, osteosarcoma, and prostate cancers [40,42,43], the in vivo effects have only been investigated in colorectal cancer [44], esophageal cancer [6], osteosarcoma [42], and prostate cancer [45,46], and the effects of fangchinoline on ovarian cancer have never been investigated. In the present study, we identified the formation of two electrostatic hydrogen bonds (strong interactions) between fangchinoline and two amino acids (Tyr246, 1.98 Å; Lys250, 3.09 Å) in Aurora A. Other weak interactions included hydrophobic alkyl–alkyl interactions, pi–alkyl interactions, hydrophobic pi–sigma interactions, and hydrophobic pi–pi interactions, which stabilize the structure of the fangchinoline and Aurora A complex. The binding and inhibitory effects of fangchinoline against Aurora A were confirmed by a CETSA and Aurora A enzymatic activity assay. In addition, fangchinoline decreased the cell viability and proliferation of ovarian cancer cells. In tumor tissue, p-Aurora A and ki67 expressions were decreased, and apoptosis was enhanced by fangchinoline. These findings suggest that the novel Aurora A inhibitor, fangchinoline, may have the potential for use in ovarian cancer therapy.

Ineffective chemo-treatment response at advanced stages is one of the main problems in ovarian cancer therapy. To treat ovarian cancer, the standard treatment is surgical resection, followed by platinum-based chemotherapy. After surgery, patients receive platinum/taxane drugs intravenously, every 21 days, for six cycles (first-line chemotherapy). However, in advanced stages (Stage III/IV), complete tumor resection is often not possible [47]. In addition, cisplatin treatment is often limited by dose-associated neurotoxicity and nephrotoxicity [48,49]. Therefore, developing new approaches to improve cisplatin efficacy could have great impacts in the fight against ovarian cancer. Cisplatin acts primarily by forming DNA adducts, causing a DNA damage response, which induces cell death [50,51]. The overexpression of Aurora A is suggested to be associated with drug resistance [52,53,54,55,56,57]. In ovarian cancer, Aurora A expression can predict platinum resistance in serous patients [58]. The combination of the Aurora A inhibitor alisertib with cisplatin increases the sensitivity to cisplatin therapy in gastric cancer [56,59]. Another inhibitor, ENMD-2076, enhances the cytotoxic effects of cisplatin in ovarian cancer [14,60]. Our study also found that cisplatin treatment increased the expression of Aurora A in OVCAR-3 cells. In summary, Aurora A inhibitors could improve the effects of cisplatin therapy in ovarian cancer. We found a synergistic effect on OVCAR-3 viability in response to combination treatment with fangchinoline and cisplatin. Fangchinoline increased the levels of cisplatin–DNA adducts and enhanced the inhibitory efficacy of cisplatin against tumor growth in a mouse ovarian cancer model. These findings indicate that fangchinoline improves the efficacy of cisplatin cancer therapy.

In conclusion, this study is the first to show that the novel Aurora A inhibitor fangchinoline exerts an enhancing effect when combined with cisplatin in ovarian cancer therapy. Additional pre-clinical studies are needed before clinical application.

## 4. Materials and Methods

### 4.1. Structure-Based VS

GOLD software is a protein-ligand docking software developed by The Cambridge Crystallographic Data Centre (Cambridge, UK), which has been validated by various researchers [61,62]. To identify potential natural products that bind the Y–W site of Aurora A, structure-based vs. was performed using GOLD 5.8.0 software [23,31,63]. A total of 2303 three-dimensional (3D) natural product structures were obtained from the Taiwan Database of Extracts and Compounds (https://tdec.kmu.edu.tw/; accessed on 4 September 2019). All structures obtained from the database were optimized using energy minimization with the MMFF94 force field in ChemBio3D software (CambridgeSoft Corporation, Waltham, MA, USA, 2014 version). The structure of Aurora A (PDB ID: 5ORL) was obtained from Protein Data Bank (https://www.rcsb.org/; accessed on 4 September 2019) [18,64]. All substrates, including co-crystal ligands, metals, and ions within the protein structure, were removed by BIOVIA Discovery Studio 2018 Visualizer (DS 2018 Visualizer; 3DEXPERIENCE Company, Waltham, MA, USA); however, co-crystallized water molecules within the protein structure were retained. Covalent hydrogen molecules and atomic charges within the protein structure were added by CHARMM force field with Momany-Rone charge using DS 2018 Visualizer software. The size of the molecular docking space was set at the extension region within 6 Å from the ligand (PDB ID: A4W)-bound location in the Y–W site of the protein structure [19]. The binding affinities between the natural products and the protein were estimated using the goldscore function in GOLD 5.8.0 software. All parameters for VS calculation were set to default except the search efficiency, which was set to 30%. Finally, docking results were simulated in DS 2018 Visualizer software.

### 4.2. Chemicals

Fangchinoline, amentoflavone, palmatine hydrochloride, sennoside B, docetaxel, bisdemethoxycurcumin, (+)-bicuculline, protopine, isoliquiritigenin, emodin, berberine, Cephalomannine, 3-(4,5-Dimethylthiazol-2-yl)-2,5-diphenyltetrazolium bromide (MTT), and dimethyl sulfoxide (DMSO) were purchased from Sigma (U.S.).

### 4.3. Cell Culture

Human ovarian cancer cell lines MDAH 2774 (RRID: CVCL_0420), SK-OV-3 (RRID: CVCL_0532), OVCAR-3 (RRID: CVCL_0465), and ES-2 (RRID: CVCL_3509) were from American Type Culture Collection (ATCC) and cultured in Dulbecco’s modified Eagle’s medium (DMEM) or Roswell Park Memorial Institute (RPMI) containing 10% of fetal bovine serum (FBS), and 5% of antibiotics (penicillin and streptomycin). Cells were incubated in an incubator at 37 °C and 5% CO_2_.

### 4.4. Cell Viability Assay

The cell viability effects of 12 candidate compounds were evaluated by MTT assay and compared against a control group (medium containing 0.1% DMSO). The cells were seeded in 96-well plates and pre-cultured for 24 h (1 × 10^5^ cells/well). The cells were then incubated for 48 h with the 12 identified compounds (at 0, 3.125, 6.25, 12.5, 25, 50, or 100 µM) dissolved in 0.1% DMSO. After 48 h, MTT solution (2.5 mg/mL in DMEM) was added to the cells and incubated for 1 h. DMSO was added to dissolve the purple crystals, and the absorbance values were measured at 575 nm using a spectrophotometer (SPECTROstar Nano; BMG LABTECH, Offenburg, Germany) [65]. Each treatment group has four biological replicates (*n* = 4). The IC_50_ values for each compound in each cell line were obtained using the results of the MTT assay and calculated using the GraphPad Prism 6 program (GraphPad Software, Inc., San Diego, CA, USA). A CI higher than 1.0 indicates an antagonistic effect, a CI equal to 1.0 indicates an additive effect, and a CI lower than 1.0 indicates a synergistic effect [66].

### 4.5. Aurora A Binding Assay

The protein-ligand binding can be evaluated by the CETSA [24,25]. This assay was developed by assessing the change of thermal stability of the protein by the binding of ligands. The binding of small molecules to a protein with high affinity leads to the conformational changes of the protein and the increase in its thermal stability. In brief, MDAH 2774 and OVCAR-3 cells were seeded in 15 cm cell culture dishes (1 × 10^6^ cells per mL) and pre-cultured for one day before the ligand treatments. Then, cells were treated with fangchinoline 0, 20, 40, 60, or 80 μM and indicated for 1 h (37 °C). After fangchinoline incubation, the cells were washed with PBS and collected in PBS (1 × 10^6^ cells/mL). Cells in tubes were subject to a 3 min heat shock (50 °C) for generating melt curves followed by rapid cooling to 25 °C. After heat treatment, Aurora A protein in cells was detected by Western blot analysis [67].

### 4.6. Aurora A Enzyme Activity Assay

Aurora A Kinase Assay kit (catalog number (#) V1931) was purchased from Promega (Madison, WI, USA). The procedures were performed following the protocol provided by Promega. After the incubations of kit reagents and fangchinoline, luminescence was detected to evaluate the Aurora A enzyme activity.

### 4.7. Immunoblotting

We loaded 50 μg of protein on 10% sodium dodecyl sulfate-polyacrylamide gel electrophoresis and then transferred it to nitrocellulose sheets (NEN Life Science Products, Inc., Boston, MA, USA) in a transfer apparatus (#1703930, Bio-Rad, Hercules, CA, USA) run at 1.2A for 3 h. After we blocked the blots in 5% nonfat skim milk in Tris-buffered saline Tween-20, the blots were incubated with Aurora A primary antibody (dilution 1:1000; I; #ab1287, Abcam, Cambridge, U.K.) or actin primary antibody (dilution 1:10000; #ab8226, Abcam) against target protein in 5% nonfat skim milk and then with secondary antibodies (dilution #14708, 1:5000 or #14709, 1:10000, respectively). After adding HRP substrates, the expression was detected using a charge-coupled device camera (ChemiDoc, Bio-Rad) and the software Image Lab 5.2.1 (Bio-Rad) [68].

### 4.8. Assessing Cisplatin–DNA Adduct Level

To investigate the level of cisplatin–DNA adduct in cancer cells, OVCAR-3 cells were treated with cisplatin (10 μM) plus fangchinoline (0, 4, or 16 μM) for 24 h. Cells were seeded in 10 cm cell culture dishes (1 × 10^6^ cells per mL) and pre-cultured for one day before the ligand treatments. Then, the cisplatin–DNA adduct levels were detected. After cells were fixed and collected, they were stained with anti-cisplatin modified DNA antibody (dilution 1:1000, #ab103261, Abcam) overnight, followed by incubated with FITC anti-rat IgG antibody (dilution 1:1000, #sc2831, Santa Cruz Biotechnology, Dallas, TX, USA) for 2 h. Fluorescence was recorded using FACS Calibur (BD Biosciences, San Jose, CA, USA) [69].

### 4.9. Animal Experiment

A total of 28 6-week-old female NOD SCID mice weighing approximately 20 g were used (BioLASCO Taiwan Co., Ltd., Taipei, Taiwan). Mice were housed in a specific pathogen-free room with temperature and humidity control (25 °C, 70% humidity) under a 12 h light/dark cycle and allowed free access to food and water. The animal care guidelines and all experimental protocols were approved by Institutional Animal Care and Use Committees (IACUC). Ovarian tumor-bearing mice were established by the subcutaneous injection of OVCAR-3 cells (1 × 10^6^/100 µL PBS/mouse), and tumors were allowed to grow to the size 50 mm^3^. After the ovarian cancer tumor model was established, all mice were randomly separated into four groups (*n* = 7): the control group, the fangchinoline group, the cisplatin group, and the cisplatin plus fangchinoline group. In the fangchinoline group, mice received fangchinoline (7 mg/kg, iv) once per week. In the cisplatin group, mice received cisplatin (3 mg/kg, iv) once per week. In the cisplatin plus fangchinoline group, mice received cisplatin (3 mg/kg, iv) and fangchinoline (7 mg/kg, iv) as separate injections once per week. All injections were performed in the morning. The first day of drug treatment was considered Day 1. The ratio of cisplatin and fangchinoline was determined based on the combination effect data. Tumor sizes were measured by using a digital caliper, and the volume was calculated using V = (length × width × height)/2 [70], and body weights were measured throughout the experiment. All mice were sacrificed on Day 21, and tumors were removed for size observation. All experimental procedures were performed in a laminar flow hood at an animal center of a Chinese Medical University in Taiwan.

### 4.10. Immunohistochemical Staining

Protein expression in tumor tissue was detected by immunohistochemical staining. In brief, tumor tissues were fixed in 10% neutral-buffered formalin and then embedded in paraffin. Paraffin-embedded tumor sections on slides were dewaxed and rehydrated by immersing the tissue in series concentrations of ethanol and xylene. After blocking for 30 min, sections were incubated with Aurora A (dilution 1:200; #PA5-97490, Invitrogen, MA, USA), p-Aurora A (dilution 1:100, #44-1210G, Invitrogen), cleaved caspase 3 (dilution 1:500, #9661, Cell Signaling Technology, Danvers, MA, USA) or Ki-67 (dilution 1:200, #9661, Cell Signaling Technology) primary antibodies for overnight and with secondary peroxidase antibodies (dilution 1:400; Cell Signaling Technology) for 1 h. Protein expressions were expressed by adding peroxidase substrate solution. Images were taken by a light microscopy Leica DM500 (×100) with the software Leica application suite (Leica, Wetzlar, Germany) [66].

### 4.11. Statistical Analysis

The statistical significance difference between groups was analyzed by student *t*-test using GraphPad Prism 6 program (GraphPad Software Inc., USA). A *p*-value lower than 0.05 was considered statistically significant.

## Figures and Tables

**Table 1:** Effects of combination cisplatin and fangchinoline treatments.

Molar Ratio (Cisplatin vs. Fangchinoline)	Combination Index (CI) Values (Cisplatin vs. Fangchinoline)
16:1	1.037 ± 0.17
4:1	0.77 ± 0.08
2:1	1.042 ± 0.046
1:1	0.513 ± 0.106
0.5:1	0.78 ± 0.15

OVCAR-3 viability was measured at 48 h, and CI values were calculated. Data are expressed as the mean ± SD (*n* = 3).

## Data Availability

All supporting data are available by contacting the corresponding authors.

## Supplementary Materials

The following are available online at https://www.mdpi.com/article/10.3390/ijms23031868/s1.

## Abbreviations

Aurora A	Aurora A kinase
VS	virtual screening
IC50	half-maximal inhibitory concentration
#	catalog number
CETSA	cellular thermal shift assay
CI	combination index
NOD SCID	non-obese diabetic severe combined immunodeficiency
GA	genetic algorithm
MTT	3-(4,5-Dimethylthiazol-2-yl)-2,5-diphenyltetrazolium bromide
DMSO	dimethyl sulfoxide
DMEM	Dulbecco’s modified Eagle’s medium
RPMI	Roswell Park Memorial Institute
FBS	fetal bovine serum
SD	standard deviation
