# Supplementary material for: Novel Aurora A Kinase Inhibitor Fangchinoline Enhances Cisplatin–DNA Adducts and Cisplatin Therapeutic Efficacy in OVCAR-3 Ovarian Cancer Cells-Derived Xenograft Model
Source: Int J Mol Sci. 2022 Feb 7;23(3):1868. doi: 10.3390/ijms23031868 (PMC8836832; doi:10.3390/ijms23031868)
Supplement: Supplementary file 1 [file ijms-23-01868-s001.zip › ijms-1575407-supplementary.pdf]

International Journal of *Molecular Sciences*

MDPI

Supplementary results

Table S1. The list of the goldscore of the 12 compounds.

No.	Name	Goldscore
1	Amentoflavone	58.41
2	Fangchinoline	57.19
3	Palmatine hydrochloride	54.95
4	Sennoside B	53.58
5	Docetaxel	53.09
6	Bisdemethoxycurcumin	51.02
7	(+)-Bicuculline	50.89
8	Protopine	50.89
9	Isoliquiritigenin	50.49
10	Emodin	50.03
11	Berberine	49.68
12	Cephalomannine	49.66
13	Aurora A ligand (PDB ID: A4W)	49.03

Table S2. The IC_{50} of the 12 compounds on ovarian cancer cell lines viability.

Compounds	IC_{50} (μM)			
	MDH2774	ES2	OVCAR3	SKOV3
Amentoflavone	99.32±0.03	-	-	78.87±0.80
Fangchinoline	8.71±0.24	25.10±0.65	9.66±0.30	11.74±1.20
Palmatine hydrochloride	51.29±0.34	-	-	59.33±0.02
Sennoside B	-	-	-	119.9±0.02
Docetaxel	24.55±0.011	50.38±0.25	40.59±0.015	21.48±0.01
Bisdemethoxycurcumin	25.97±0.53	45.47±0.50	24.95±0.74	25.14±1.90
(+)-Bicuculline	-	-	-	-
Protopine	-	-	25.44±0.68	26.12±0.49
Isoliquiritigenin	50.36±0.48	51.46±0.32	25.79±1.21	25.98±0.44
Emodin	34.32±0.021	50.23±2.09	22.64±0.030	34.7±0.02
Berberine	12.78±1.24	87.6±0.02	20.56±0.021	39.38±0.02
Cephalomannine	41.72±0.01	-	41.78±0.013	10.79±0.01

IC_{50}: half maximal inhibitory concentration.
Data are presented as the mean ± SD (standard deviation).
